# Diverse Developmental Disorders from The One Ring: Distinct Molecular Pathways Underlie the Cohesinopathies

**DOI:** 10.3389/fgene.2012.00171

**Published:** 2012-09-12

**Authors:** Julia A. Horsfield, Cristin G. Print, Maren Mönnich

**Affiliations:** ^1^Department of Pathology, Dunedin School of Medicine, The University of OtagoDunedin, New Zealand; ^2^Department of Molecular Medicine and Pathology, School of Medical Sciences, Bioinformatics Institute, The University of AucklandAuckland, New Zealand

**Keywords:** cohesin, gene expression regulation, animal models, CdLS, RBS

## Abstract

The multi-subunit protein complex, cohesin, is responsible for sister chromatid cohesion during cell division. The interaction of cohesin with DNA is controlled by a number of additional regulatory proteins. Mutations in cohesin, or its regulators, cause a spectrum of human developmental syndromes known as the “cohesinopathies.” Cohesinopathy disorders include Cornelia de Lange Syndrome and Roberts Syndrome. The discovery of novel roles for chromatid cohesion proteins in regulating gene expression led to the idea that cohesinopathies are caused by dysregulation of multiple genes downstream of mutations in cohesion proteins. Consistent with this idea, *Drosophila*, mouse, and zebrafish cohesinopathy models all show altered expression of developmental genes. However, there appears to be incomplete overlap among dysregulated genes downstream of mutations in different components of the cohesion apparatus. This is surprising because mutations in all cohesion proteins would be predicted to affect cohesin’s roles in cell division and gene expression in similar ways. Here we review the differences and similarities between genetic pathways downstream of components of the cohesion apparatus, and discuss how such differences might arise, and contribute to the spectrum of cohesinopathy disorders. We propose that mutations in different elements of the cohesion apparatus have distinct developmental outcomes that can be explained by sometimes subtly different molecular effects.

## Introduction

The cohesin complex and proteins that regulate its interaction with chromatin have multiple roles in cell division, DNA damage repair, gene transcription, and chromosome architecture. Proteins that make up the cohesin complex have been characterized in several model systems (see Table 1). The mechanics of cell division has been well researched for decades, and the identity of the chromosome cohesion proteins that hold together sister chromatids after S phase and prior to mitosis has been known for 15 years (Guacci et al., 1997; Michaelis et al., 1997). Consequently, sister chromatid cohesion remains the best-characterized role for the cohesin complex and its regulators.

**Table 1:** **Nomenclature and function of cohesin subunits and cohesin regulators**.

Chromosome cohesion regulator	*S. cerevisiae*	*S. pombe*	*D. melanogaster*	*X. laevis*	*D. rerio*	*H. sapiens*	Function
SMC subunits	Smc1	Psm1	SMC1	smc1a^1^	Smc1al, Smc1a^2^	SMC1A	Core cohesin subunit
				smc1b^1^	Smc1b	SMC1B	Cohesin subunit (meiosis)
	Smc3	Psm3	Cap/SMC3	smc3/cspg6^1^	Smc3	SMC3/CSPG6/Bamacan	Core cohesin subunit
α-Kleisin subunit	Mcd1/Scc1	Rad21	Vtd/Rad21	rad21/mcd1/nxp1/scc1^1^	Rad21a, Rad21b^2^	RAD21	Core cohesin subunit
	Rec8	Rec8	C(2)M	rec8	Rec8/zgc: 136888^1^^,^^3^	REC8	Cohesin subunit (meiosis)
	*–*	–	–		Rad21l1	RAD21L1/RAD21L	
Stromalin/SA subunit		Psc3	SA (stromalin)	stag1/sa1	Stag1^1^^,^^3^	STAG1/SA1/SCC3A	Cohesin subunit
			SA2 (stromalin-2)	stag2/sa2^1^	Stag2^1^^,^^3^	STAG2/SA2/SCC3B	
–		Rec11	–	stag3/sa3^1^	Stag3l3^1^	STAG3/SA3	Cohesin subunit (meiosis)
Interactors of α-kleisin and SA	Pds5	Pds5	Pds5	pds5a	Pds5a/zgc: 66331	PDS5A	Balancing cohesion establishment with cohesin dissociation
				pds5b/as3/aprin^1^	Pds5b^1^	PDS5B/APRIN/AS3	
	?	?	Dmt (Dalmatian)	cdca5/sororin^1^	Cdca5	CDCA5/SORORIN	
	Rad61/Wpl1	Wapl	Wapl	wapal	Wapl/KIAA 0261^1^^,^^3^	WAPAL/WAPL	
Kollerin	Scc2	Mis4	Nipped-B	nipbl/scc2/delangin	Nipbla/Scc2a, Nipblb/Scc2b	NIPBL/SCC2/DELANGIN	Cohesin loading
	Scc4	Ssl3		mau2/scc4^1^	Mau2/zgc: 112338^1^	MAU2/SCC4	
Cohesin acetyl transferase (CoAT)	Eco1/Ctf7	Eso1	Eco/Deco San	esco1 esco2/rbs/efo2^1^	Esco1^1^ Esco2	ESCO1 ESCO2	Establishment of cohesion
Cohesin deacetylase (CoDAC)	Hos1	?	?	hdac8	Hdac8	HDAC8	Recycling of cohesin

*^1^Predicted/*in silico* annotated only*.

*^2^No functional data available*.

*^3^Duplicated (EnsemblZv9, release 68)*.

*?, protein not yet identified*.

The first evidence that a transcriptional function existed for chromosome cohesion proteins emerged in 1999, when the *Nipped-B* gene was identified in a genetic screen for modifiers of long-range enhancer-promoter communication regulating *cut* gene expression in the *Drosophila* wing margin (Rollins et al., 1999). Further evidence that gene transcription isone of cohesin’s crucial functions unfolded over the following years, and included a role for Scc1 in mating-type silencing in yeast (Lau et al., 2002), a transcriptional co-activation function for SA in mammalian cell lines (Lara-Pezzi et al., 2004), and complex long-range regulation of *cut* gene expression resulting from cohesin and *Nipped-B* *Drosophila* mutants (Rollins et al., 2004). Interest in the transcription function of cohesion proteins heightened when heterozygous mutations *NIPBL*, the human homolog of *Nipped-B*, were found to cause the human developmental disease, Cornelia de Lange Syndrome (CdLS; OMIM 122470; Krantz et al., 2004; Tonkin et al., 2004). Additional mutations causing CdLS were found in the cohesin subunits SMC1 (Musio et al., 2006; Deardorff et al., 2007) and SMC3 (Deardorff et al., 2007). Furthermore, homozygous mutations in *ESCO2*, which encodes a cohesion acetyltransferase (CoAT; Nasmyth, 2011; Higashi et al., 2012), were found to underlie a second human disorder, Robert’s Syndrome (RBS; OMIM 268300; Vega et al., 2005). More recently, mutations in RAD21 have been found to cause a related developmental disorder that partially overlaps with CdLS (Deardorff et al., 2012b).

After the causative genes for CdLS and RBS were found, a flood of new results in vertebrates, from fish (Horsfield et al., 2007; Muto et al., 2011), mouse (Zhang et al., 2007, 2009; Kawauchi et al., 2009), and human cell lines (Liu et al., 2009), supported the notion that these syndromes could be caused by dysregulated expression of multiple developmental genes. This suggested that cohesin-related developmental disorders have related pathologies, and led to use of the term “cohesinopathies” to describe these disorders (Liu and Krantz, 2008; McNairn and Gerton, 2008).

Although the idea that cohesinopathies have a common causal basis in dysregulated gene expression is a popular one, it is clear that the output of gene regulation is different for each disorder. Human syndromes caused by *NIPBL*, *SMC1*, *SMC3*, *RAD21*, and *ESCO2* mutations share common features but appear to be clinically distinct. Here we revisit the theory that cohesinopathies result from dysregulated gene expression, and raise the question of whether subunits contributing to cohesin or its regulation can interact separately with distinct pathways leading to diverse phenotypic consequences.

## Overview of Cohesin Structure and Function

**Figure 1:**
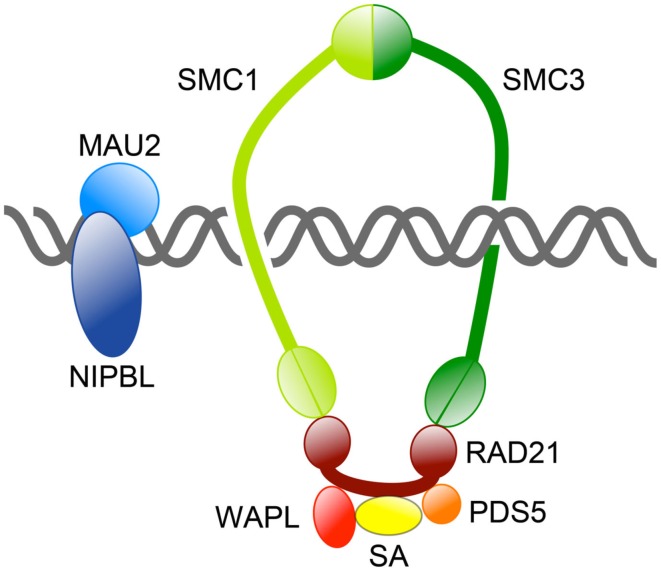
**Overview of the cohesin complex and its associated proteins**. The cohesin complex consists of four core subunits: SMC1, SMC3, RAD21, and SA. Together these subunits form a large ring capable of topologically encircling DNA strands. Other proteins regulate cohesin’s binding to DNA and its residency there. The NIPBL/MAU2 dimer loads cohesin onto DNA, whereas WAPL/PDS5 release cohesin from chromosomes by opening the SMC3-RAD21 interface.

The mitotic cohesin complex comprises two structural maintenance of chromosomes (SMC) subunits Smc1 and Smc3, which associate to form a tripartite ring incorporating an α-kleisin subunit, Mcd1/Scc1/Rad21. Smc1 and Smc3 are large rod-shaped proteins that dimerize at one end to form a “hinge” domain, and also interact at the other end via ATP-binding “heads,” which in turn interact with the α-kleisin subunit (Figure 1). The α-kleisin interacts with additional subunits Scc3/Stromalin (SA), Pds5, and Wapl (Nasmyth, 2011; see Table 1). The formation of cohesin subunits into a large ring structure led to the theory that cohesin topologically entraps sister chromatids inside a single ring (Haering et al., 2008). Alternative models have been proposed for how cohesin physically holds two molecules of DNA together (Huang et al., 2005; Zhang et al., 2008b; Skibbens, 2010), although most are not compatible with the single ring theory (reviewed in Nasmyth, 2011).

**Figure 2:**
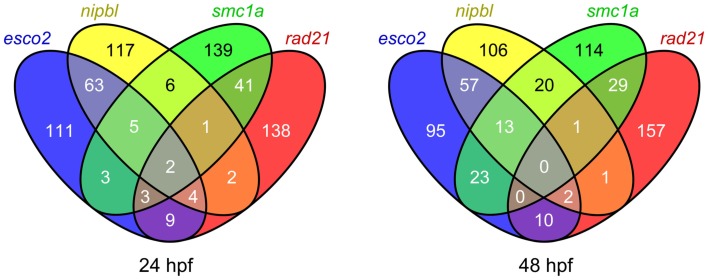
**Comparison of the top 200 affected probe sets in zebrafish embryos depleted of different cohesin subunits**. Venn diagrams showing the overlap of the top 200 probe sets affected in zebrafish *esco2* and *nipbl* morphants, and *smc1a^hi1113a^* and *rad21^nz171^* mutants at 24 and 48 hpf (*q* value < 0.05).

The many functions of cohesin have been well described in recent reviews (Hirano, 2006; Nasmyth and Haering, 2009; Carretero et al., 2010; Nasmyth, 2011; Rhodes et al., 2011; Mehta et al., 2012). Cohesin turnover, recycling, loading onto chromosomes and residency there is controlled by several other proteins (Figure 2; Table 1). It was recently proposed that cohesin is loaded and unloaded from chromosomes by a “dual gate” mechanism (Nasmyth, 2011). The cohesin loading complex containing Scc2 (Nipped-B in *Drosophila* and NIPBL in human) and Scc4/MAU2, recently dubbed “kollerin” (Nasmyth, 2011), is responsible for loading cohesin onto chromosomes in G1 phase in yeast, and telophase in most other organisms. Kollerin directly loads cohesin onto the pre-replication complex (pre-RC) on chromatin *in vitro* in *Xenopus* extracts (Bermudez et al., 2012), indicating that it is likely to be necessary and sufficient for cohesin loading. Kollerin likely facilitates cohesin loading by enabling the transient opening of the Smc1-Smc3 hinge domains (Figure 1; Nasmyth, 2011). An opposing unloading activity is mediated by “releasin,” a cohesion disestablishment complex containing Pds5 and Wapl that interacts with SA to unlock the cohesin ring (Gandhi et al., 2006; Kueng et al., 2006; Shintomi and Hirano, 2009). Releasin allows exit of DNA via the Smc1-Smc3 head domains by opening the Smc3-kleisin interface. In theory, cohesin snaps onto DNA via opening of the hinge domains, and exits DNA via opening the ring at the opposite end (Nasmyth, 2011).

Once loaded onto chromosomes, cohesin binds DNA with variable modes of stability (Gerlich et al., 2006; Gause et al., 2010) and is mobile, having the ability to translocate along chromosome arms (Lengronne et al., 2004; Hu et al., 2011), or readily detach via interaction with releasin. However during S phase, cohesin becomes stably bound for long enough to fulfill its function in sister chromatid cohesion. Stabilization of cohesin binding happens during the process of DNA replication (Skibbens et al., 1999; Kenna and Skibbens, 2003; Moldovan et al., 2006), and is mediated via acetylation of Smc3 by cohesin acetyl transferase (CoAT; Nasmyth, 2011). The known CoATs for Smc3 are Ctf7/Eco1 (yeast), or Esco1/2 (vertebrates; Skibbens et al., 1999; Ivanov et al., 2002; Hou and Zou, 2005).

CoAT-mediated acetylation of Smc3 generates the cohesive form of cohesin that holds together the sister chromatids from G2 until M phase (Ben-Shahar et al., 2008; Unal et al., 2008; Zhang et al., 2008a). In humans, both ESCO1 and ESCO2 CoATs are necessary for proper sister chromatid cohesion (Hou and Zou, 2005). However, it appears that ESCO2 CoAT is primarily required for cohesion in heterochromatic regions, and RBS patients who lack ESCO2 exhibit heterochromatin repulsion and precocious sister chromatid separation, particularly at centromeric regions (Vega et al., 2005). In human and *Drosophila* (but not yeast), the Sororin protein is additionally required to establish and maintain cohesion (Rankin et al., 2005; Schmitz et al., 2007; Nishiyama et al., 2010).

Once cohesion has been established in G2, cohesion-promoting and cohesin-releasing activities compete during chromosome condensation in prophase. The releasing activity that removes cohesin from chromosomes prevails along chromosome arms in a process known as the “prophase pathway,” which involves phosphorylation of SA1/2 by Polo-like kinase (Plk) and Aurora B (Losada et al., 2002; Hauf et al., 2005) and complexing of SA and RAD21 by releasin (Gandhi et al., 2006; Kueng et al., 2006; Shintomi and Hirano, 2009). In the competing “establishment” activity, Sororin and CoAT function to antagonize releasin activity (Rowland et al., 2009; Sutani et al., 2009; Lafont et al., 2010; Nishiyama et al., 2010; Nasmyth, 2011) by a mechanism that also requires Pds5 (Vaur et al., 2012), and the phosphatase Ssu72 promotes cohesion by countering the phosphorylation of SA1/2 (Hauf et al., 2005; Kim et al., 2010b). By metaphase, most cohesin has been removed from chromosome arms, and the remaining, primarily centromeric cohesin, is protected from removal by Shugoshin (Wang and Dai, 2005).

At Anaphase, the remaining cohesin rings are opened, allowing chromosomes to separate (Craig and Choo, 2005). APC-mediated degradation of Securin (Salah and Nasmyth, 2000) releases the protease Separase, which cleaves the Rad21 subunit of cohesin (Waizenegger et al., 2000, 2002; Hornig et al., 2002). After telophase, Smc complexes can be recycled and reloaded onto chromatin. An important requirement for cohesin recycling is deacetylation of Smc3 by the class I histone deacetylase Hos1 (yeast) or HDAC8 (human; Beckouet et al., 2010; Borges et al., 2010; Xiong et al., 2010; Deardorff et al., 2012a). Thus, Smc3 deacetylation by Hos1 opposes Esco2’s acetylation activity.

Cohesin has a further important role in DNA double strand break repair (reviewed in Dorsett and Strom, 2012; Wu and Yu, 2012). To effect double strand break repair, the cohesive form of cohesin must be established at the location of the break (Ball and Yokomori, 2008). Stabilization of cohesin at double strand breaks in budding yeast depends on acetylation of the Rad21/Mcd1p subunit by Eco1p, plus antagonism of the releasin complex containing Wpl (Heidinger-Pauli et al., 2009). Cohesin is recruited *de novo* at double strand breaks in G2 phase (Strom et al., 2007) in a Scc2/kollerin-dependent manner (Strom et al., 2004), and in vertebrates, this association also involves another SMC complex: the Smc5/6 complex (Strom and Sjogren, 2007; De Piccoli et al., 2009).

Other molecular events contribute to cohesin function in DSB repair. In budding yeast, it was shown that the phosphorylation of Mcd1p (Rad21) through ATR and Chk1 pathway is important for cohesion and DSB repair (Heidinger-Pauli et al., 2008). In human cells, cohesive cohesin at DSBs also depends on the pro-establishment activity of Sororin (Schmitz et al., 2007). Cohesin, but not chromosome cohesion, is required for activation of G1, intra-S, and G2–M DNA damage checkpoints (Jessberger, 2009; Watrin and Peters, 2009). In cancer cells, cohesin binding through the genome is reinforced following ionizing radiation (IR), in a process that requires ATM and SMC3 phosphorylation, and SMC3 acetylation by ESCO1. Both ESCO1 and SMC3 acetylation are required for intra-S phase checkpoint and cellular survival after IR (Kim et al., 2010a).

## Cohesin and Mechanisms of Gene Transcription

Despite good evidence that cohesin regulates gene expression directly and independently of cell division (Pauli et al., 2010; Dorsett, 2011), the mechanism(s) of transcriptional regulation by cohesin are not well understood. Cohesin binds to many sites throughout the genome, sometimes in combination with the CCCTC-binding factor (CTCF) insulator protein, which is known to mediate chromatin loop formation (Gondor and Ohlsson, 2008). Previous studies demonstrated that cohesin colocalizes with CTCF along chromosome arms, and is likely to cooperate with this protein in the regulation of gene expression or chromatin structure (Parelho et al., 2008; Rubio et al., 2008; Wendt et al., 2008). As well as CTCF, cohesin colocates genome-wide with other transcriptional regulators, such as estrogen receptor-α (Schmidt et al., 2010), and Mediator (Kagey et al., 2010) in a cell type-specific manner. Likely in combination with other factors, cohesin selectively binds genes with paused RNA polymerase. Although it is not involved in RNA polymerase pausing itself, cohesin can regulate transcription by determining the amount of elongating RNA polymerase on genes (Fay et al., 2011).

Regulation of many genes by cohesin appears to involve the three-dimensional (3D) organization of chromatin (Merkenschlager, 2010; Dorsett, 2011). A direct role for cohesin in chromatin looping has been demonstrated for several loci (Hadjur et al., 2009; Mishiro et al., 2009; Nativio et al., 2009; Hou et al., 2010; Chien et al., 2011; Kim et al., 2011) by studies showing that long-range interactions between regulatory sequences are reduced by cohesin knockdown. It is likely that cohesin regulates spatiotemporal gene expression in combination with diverse tissue-specific transcription factors, and by distinct modes of transcription regulation (Dorsett, 2011).

## The Human Cohesinopathies

An overlapping spectrum of human syndromes can be attributed to mutations in cohesin subunits, or regulators of cohesin loading and unloading from chromosomes. The best known cohesinopathy is CdLS (OMIM 122470) also termed Brachmann de Lange syndrome (BdLS), a broad spectrum disorder with multiple developmental and cognitive abnormalities (de Lange, 1933; Opitz, 1985; Ireland et al., 1993; Jackson et al., 1993). CdLS patients are small in size and have a characteristic facial appearance, including arched eyebrows, hirsutism, synophrys, ptosis, long eyelashes, an upturned nose, a long philtrum, thin upper lip, and micrognathia. Developmental anomalies range from mild to severe, with more severe cases having upper limb truncations or limb differences. CdLS patients also frequently present with hearing loss, gastrointestinal defects, pyloric stenosis, genital abnormality, congenital diaphragmatic hernias, cardiac septal defects, and autistic behaviors (Jackson et al., 1993). All patients within the CdLS spectrum have neurodevelopmental delay and highly variable mental retardation (Deardorff et al., 2007).

More than half of CdLS cases (∼65%) are dominantly inherited, and caused by mutations in the *NIPBL* gene (OMIM 608667; Krantz et al., 2004; Tonkin et al., 2004), which encodes a crucial component of kollerin. Heterozygous truncating or non-sense *NIPBL* mutations are haploinsufficient, and strikingly, NIPBL protein levels need only be reduced by 15–30% to give rise to a CdLS phenotype (Krantz et al., 2004; Tonkin et al., 2004). This implies that the remaining intact *NIPBL* allele is upregulated in an attempt to compensate, and also that certain cell types and/or developmental processes are exquisitely sensitive to the levels of NIPBL. Missense mutations in *NIPBL* were also identified that may interfere with the interaction of NIPBL with its partner, MAU2, or other proteins (Braunholz et al., 2012).

Mutations in *SMC1A* (OMIM 300040) and *SMC3* (OMIM 606062) also give rise to syndromes that fall within the CdLS spectrum, and account for about 5% of CdLS cases (Musio et al., 2006; Deardorff et al., 2007; Mannini et al., 2010). *SMC* mutations are heterozygous missense mutations and are thought to interfere with the structure of the SMC subunits such that functional interactions of the cohesin complex are disturbed, causing the disease pathology (Deardorff et al., 2007). In some cases missense mutations were shown to interfere with cohesin binding to DNA (Revenkova et al., 2009). Human developmental phenotypes resulting from *SMC* mutations are inclined to be milder than for *NIPBL* mutations; these individuals have fuller eyebrows and a prominence of the nasal bridge, with fewer structural abnormalities; however, all patients had some degree of mental retardation (Deardorff et al., 2007; Rohatgi et al., 2010). This suggests that brain development is particularly sensitive to disruption of SMC subunits.

*RAD21* (OMIM 606462) mutations also cause a cohesinopathy syndrome (Deardorff et al., 2012b). Heterozygous deletions of *RAD21* and missense mutations, which included a dominant interfering mutation and one with essentially no function, gave rise to developmental anomalies with some overlap with CdLS. Patients with *RAD21* mutations have an even milder phenotype than those with *SMC* mutations. They have some divergence in the facial features and, most notably, they have extremely mild cognitive and physical abnormalities (Deardorff et al., 2012b). Consistent with RAD21 having a role in DNA damage response, lymphoblastoid cell lines from patients with RAD21 mutations exhibited radiation sensitivity. A gene transcription assay in zebrafish showed that *RAD21* missense mutations present in patients are not competent for proper regulation of gene expression (Deardorff et al., 2012b).

Homozygous recessive mutations in the *ESCO2* gene, which encodes a CoAT, cause another cohesinopathy, RBS (OMIM 268300; Schule et al., 2005; Vega et al., 2005, 2010; Gordillo et al., 2008). RBS is characterized by mild to severe growth deficiency, limb malformations (in particular, symmetric tetraphocomelia), multiple craniofacial abnormalities including cleft lip and/or cleft palate, microcephaly, and mental retardation. Mortality is high among severely affected pregnancies and newborns (Gordillo et al., 1993). A milder disorder with less marked limb reduction and survival to adulthood is known as SC phocomelia, but since both disorders arise from ESCO2 mutations with no apparent genotype/phenotype correlation (Schule et al., 2005; Vega et al., 2010), it has been proposed all *ESCO2* mutations be referred to as RBS (Vega et al., 2010). Unlike CdLS, cells from RBS patients exhibit precocious sister chromatid separation, particularly at heterochromatic regions of the chromosomes (Schule et al., 2005; Vega et al., 2005) leading to mitotic defects, lagging chromosomes, aneuploidy, and micronuclei formation. The acetyltransferase activity of ESCO2 appears to be crucial, since mutations in this domain are sufficient for the pathogenesis of RBS (Gordillo et al., 2008). While RBS features overlap with those of CdLS, there are appreciable differences. Whether gene regulation downstream of ESCO2 is responsible for RBS pathology is still under debate.

The wide spectrum of human developmental phenotypes owing to cohesin mutations characterized to date indicate that although these disorders have many features in common, there are also distinct differences. Gene expression and molecular studies in cells and in animal models have helped to uncover the common and divergent pathways that lie downstream of cohesinopathy mutations.

## Characterization of Cohesinopathy Mutations Reveals that Distinct Pathways are Affected by Different Cohesinopathy Mutations

A comparison of the consequences of knocking down cohesin or its regulators in different animal model systems indicates there are a wide variety of outcomes for cell biology and gene expression. For mutations causing CdLS and similar cohesinopathies, it seems likely that specific developmental pathways are regulated downstream of the causative gene mutations. Several groups have conducted analyses of gene expression downstream of cohesinopathy mutations.

For some genes, it seems likely that small changes in the dose of cohesin or its regulators could have a large impact on transcription. In *Drosophila*, cohesin and Nipped-B bind to actively transcribed regions of the genome and are excluded from regions of polycomb group (PcG) silencing (Misulovin et al., 2008). For the rare genes where cohesin binding overlaps with PcG-mediated methylation of lysine 27 on histone 3 (H3K27me3), expression of those genes is hypersensitive to cohesin dose (Schaaf et al., 2009). In addition, cohesin ablation in post-mitotic neurons in the *Drosophila* mushroom body (Pauli et al., 2008; Schuldiner et al., 2008), or salivary glands (Pauli et al., 2010) affected the expression of specific loci including the gene encoding the ecdysone receptor. This suggests that some genes, perhaps in specific cell types, may dramatically change their transcriptional activity in response to a slight alteration of cohesin dose.

Intriguingly, it seems that the transcriptional response of some genes to cohesin or Nipped-B depletion is biphasic, and depends on the degree to which these proteins are depleted (Schaaf et al., 2009). The *Enhancer of split* gene complex (E(spl)-C) in *Drosophila* is exquisitely responsive to Rad21 and Nipped-B levels. Furthermore, when mRNA encoding these proteins is depleted in BG3 cells, the direction in which some E(spl)-C are regulated depends on the length of time of RNAi treatment, and the degree of Rad21 or Nipped-B knockdown. For example, E(spl)-C transcripts decrease after 3 days of Nipped-B RNAi, but increase by day 6 (Schaaf et al., 2009). These findings have implications for genome-wide gene expression studies in cohesinopathy models. Which genes are altered in expression is likely to depend on tissue type, developmental stage and degree to which cohesinopathy gene function has been knocked down.

On the other hand, loss of Nipbl also appears to result in low (≤2) fold changes in the expression of a great many genes. Liu et al. (2009) analyzed gene expression and genome-wide binding of cohesin in lymphoblastoid cell lines from CdLS probands with mutations in *NIPBL* or in the cohesin subunit *SMC1A*, and found that ∼1500 genes (FDR ≤ 0.05) were dysregulated compared with controls. Dysregulated gene expression in the mutant cell lines was conserved, and correlated with disease severity and cohesin binding at misexpressed genes (Liu et al., 2009). Significantly, a panel of 23 genes could differentiate *NIPBL* mutations from *SMC1A* and *ESCO2* mutations indicating that *NIPBL* mutations have a distinguishable effect on gene expression.

Heterozygous mice carrying a gene-trap insertion into the *Nipbl* locus show many features overlapping with CdLS, and microarray analyses indicated that reducing Nipbl dose resulted in small changes in expression of a great many genes. These mice also had severe developmental phenotypes, including craniofacial dysmorphology and heart defects, resembling CdLS. Of note was the altered expression of genes involved in fat metabolism, which could account for the lean habitus observed in mice and in CdLS patients (Kawauchi et al., 2009).

Mice with mutations in *Pds5a* and *5b* have also been generated. Mice homozygous null for *Pds5b* died shortly after birth, with multiple congenital anomalies, including heart defects, cleft palate, skeletal defects, gut defects, abnormal migration and axonal projections of sympathetic neurons, and germ cell depletion (Zhang et al., 2007). Mice null for *Pds5a* exhibit many of the same multiple abnormalities that were previously observed in *Pds5b*-deficient mice, plus additional abnormalities including renal agenesis (Zhang et al., 2009). Elimination of both Pds5a and 5b gave an additional lens phenotype not observed in single null mice, and resulted in embryonic lethality (Zhang et al., 2009). Gene expression studies in the Pds5 mice have not been published.

Most recently, significant knowledge about cohesin function was gained by generating mice deficient for cohesin subunit SA1 (Cuadrado et al., 2012; Remeseiro et al., 2012a,b). Loss of SA1 results in embryonic lethality, and heterozygous animals have shorter lifespan and increased aneuploidy as a result of chromosome segregation defects. Segregation defects arose from compromised telomere replication, which requires cohesion mediated specifically by cohesin-SA1. The resulting aneuploidy in *SA1* heterozygotes is thought to lead to early onset of tumorigenesis in these animals (Remeseiro et al., 2012a).

Interestingly, gene expression and genome-wide distribution of cohesin binding are dramatically altered in *SA1* null mice, with important implications for CdLS. Location of cohesin to gene promoters and CTCF binding sites appears to depend on SA1. Furthermore, SA1 ablation led to altered cohesin binding at particular gene clusters accompanied by dysregulation of their transcription (Remeseiro et al., 2012b). These studies highlight the function of SA1 in multiple processes, and identify a key transcriptional role that is distinct from the function of SA2 in centromeric chromosome cohesion.

Zebrafish models have also shed light on the role of cohesin and Nipbl in gene expression. In fact, the first published evidence that cohesin regulates gene expression in a vertebrate model system came from a forward genetic screen in zebrafish. This screen identified the *Rad21* subunit as a tissue-specific regulator of *runx1*, which encodes a hematopoietic transcription factor (Horsfield et al., 2007). In *rad21* mutants at 12 h post-fertilization (hpf), *runx1* expression was retained in Rohon–Beard neurons, but was absent from a discrete population of cells in the hematopoietic mesoderm. Importantly, the hematopoietic mesoderm precursor cell population was still present in mutants, and expressed the dimerization partner for Runx1, *cbfb*, although not *runx1* itself. Cohesin probably targets other *runx* genes in a cell type-specific manner, since *rad21* mutants also lacked expression of *runx3* in Rohon–Beard neurons and the lateral line primordia (Horsfield et al., 2007). Unfortunately, the onset of *runx2* expression (∼48 hpf) in zebrafish embryos is too late to determine its involvement, since *rad21* mutants arrest in development at 35 hpf. Like in *Drosophila*, cohesin is likely to regulate expression of genes in zebrafish brain; cohesin subunits are expressed in non-proliferating neurons of zebrafish brain implying a non-cell cycle role for cohesin in this tissue (Monnich et al., 2009).

A zebrafish model of *NIPBL*-mediated CdLS revealed much about the multifactorial origins of this developmental syndrome. Zebrafish have two copies of the *nipbl* gene, and depletion of both versions by morpholino oligonucleotides to create “morphants” also led to small-scale dysregulation of a large number of genes in early embryogenesis (up to 6 hpf; Muto et al., 2011). Because gene expression changes were measured at early gastrula stages it is likely that many are directly caused by reduced Nipbl function rather than by secondary effects. Interestingly, genes involved in endoderm development and left-right axial patterning including *sox17* and *foxa2*, were specifically downregulated in endoderm. Dysregulation of the endoderm-specifying hierarchy of Sox32, Sox17, and Foxa2 by Nipbl depletion is likely to contribute to the heart looping defects and gut tube defects observed at later stages in Nipbl-depleted zebrafish embryos (Muto et al., 2011). The zebrafish pathologies recapitulate heart and gastrointestinal tract abnormalities observed in CdLS, thereby allowing insight into the etiology of CdLS developmental defects.

Our own group conducted Affymetrix microarray analyses at a later stages (24 and 48 hpf) of zebrafish development in *rad21* mutants (Rhodes et al., 2010), *esco2* morphants (Monnich et al., 2011), *smc1a* mutants (available as part of an insertion mutant collection; Amsterdam et al., 2004) and *nipbl* morphants (Maren Mönnich, Cristin G. Print, Julia A. Horsfield, unpublished data). Interestingly, we found that the *eomes* gene, a master regulator of endoderm formation, is consistently downregulated in *rad21* and *smc1a* mutants, and *nipbl* morphants (FDR < 0.02), supporting a role for cohesin and Nipbl in endoderm formation. *Eomes* expression is regulated by pluripotency factors Nanog, Oct4, and Sox2 (Teo et al., 2011), all of which are transcriptional targets of cohesin and Nipbl in embryonic stem cells (Kagey et al., 2010). It is enticing to speculate that cohesin and Nipbl could participate in the initial specification of germ layers from stem cell precursors through modulating the expression of pluripotency factors.

We expected our microarray analyses of zebrafish cohesinopathy mutants and morphants to result in similar lists of up- or downregulated genes, since embryos were analyzed at similar stages and cohesinopathy genes would be predicted to have similar roles in gene expression. Therefore we were surprised to find only modest overlap between regulated gene sets (example in Figure 2).

Strikingly, a comparison of *rad21* mutant microarray data with *esco2* morphant microarray data revealed that there is scant overlap between genes regulated downstream of these mutations (Monnich et al., 2011). For example, the *myca* gene, which is downregulated in *rad21* zebrafish mutants and other species as well, is actually slightly upregulated in *esco2* morphants. Most of the genes regulated downstream of *esco2* are involved in cell proliferation or apoptosis, whereas many genes affected by the *rad21* mutation are developmental regulators (Monnich et al., 2011). What could be the reason for these differences? We concluded that while Esco2 and Rad21 have related roles in sister chromatid cohesion, they do not have the same input into the regulation of gene expression. We found that although *esco2* depletion has mild effects on neural crest cell migration, it does not induce patterning defects. Instead, even modest *esco2* depletion results in robust activation of caspases, *p53/mdm2* upregulation, and massive cell death (Monnich et al., 2011). Loss of jaw elements and fin stunting in *esco2* morphants, which resemble RBS features, are therefore likely to be due to insufficient cells to contribute to the affected structures. In agreement with results from a conditional mouse knockout of *Esco2* (Whelan et al., 2012b), it appears that developmental defects observed in *esco2* morphant zebrafish arise from problems with cell survival rather than dysregulation of developmental genes.

Our microarray data of *nipbl* morphants was conducted under very mild knock down conditions of both *nipbl* genes at 24 and 48 hpf (Table 2; Maren Mönnich, Cristin G. Print, Julia A. Horsfield, unpublished data). We observed regulation of different sets of genes than those found by Muto et al. (2011) at the earlier timepoint of 6 hpf, which is not unexpected due the different developmental stage at which embryos were analyzed. We did not find any Gene Ontology categories of significance other than elevated expression of a network of genes related to p53. It is possible that degree of *nipbl* gene knockdown could also contribute to differences observed in regulated genes as discussed above, since at least some gene expression is likely to be sensitive to the dose of Nipbl protein (Schaaf et al., 2009).

Many genes that have altered regulation in response to depletion of cohesinopathy genes are different, raising the possibility that cohesin subunits and regulators have different functions in various pathways. However, genome-wide analyses of gene expression identified some commonly regulated pathways/genes such as those involved in endoderm development (*eomes*, *sox17*, *foxa3*), the *myc* transcription factor (except in *esco2* morphants), and downstream effectors of Notch signaling such as *hey1*, *her4.2*, and *ascl1*.

## Common Pathways Regulated by Cohesinopathy Genes

Despite varying outcomes for gene expression and development identified using animal models of the cohesinopathies, some pathways seem more likely to be affected than others downstream of cohesinopathy genes. Common themes of pathways regulated by cohesinopathy genes are outlined below.

### Growth, metabolism, and pluripotency

Perhaps not surprisingly, several studies have found links between cohesin and its regulators, and the control of pathways that underpin cell growth and proliferation. Somewhat more surprisingly, the level at which cohesin regulates growth and metabolism includes transcriptional control of specific gene targets. For example, the *Myc* oncogene is positively regulated by Nipbl and all cohesin subunits investigated to date (Misulovin et al., 2008; Kawauchi et al., 2009; Liu et al., 2009; Rhodes et al., 2010; Remeseiro et al., 2012b). Myc is a pluripotency factor, and it is probably significant that genes encoding other pluripotency factors Oct4, Nanog, and Sox2, are also bound and regulated by cohesin (Kagey et al., 2010; Nitzsche et al., 2011). Interestingly, pluripotency factors, e.g., Oct4 (Kim et al., 2011) and Nanog (Nitzsche et al., 2011) in turn appear to combine with cohesin to both positively and negatively regulate other target genes. These findings raise the interesting possibility that cohesin-mediated transcription is pivotal to cell fate decisions that determine the balance between pluripotency and differentiation (Dorsett, 2010).

Cohesinopathy genes regulate other growth pathways as well. In yeast, cohesinopathy mutations, including an Eco1RBS mutation (W216G), block transcription of ribosomal RNA genes thereby directly influencing ribosome biogenesis, protein translation and the cell’s ability to grow (Bose et al., 2012). This finding links cohesin function to metabolism and growth through a role in rDNA transcription and translation regulation. Since Myc, a transcriptional target of cohesin, also regulates ribosome biogenesis (Eilers and Eisenman, 2008), cohesin appears to be a central regulator of growth by transcriptional control of multiple pathways. In *Nipbl*^+/−^ mice, genes controlling fat metabolism are dysregulated (Kawauchi et al., 2009), indicating a direct involvement in regulation of another metabolic pathway. Consistent with dysregulated growth and metabolism, CdLS patients are small and lean (Liu and Krantz, 2009). It is possible that many of the large number of dysregulated genes in CdLS are targets of MYC, which regulates 10–20% of genes in the genome.

Transcriptional regulation of cell growth and proliferation pathways by cohesin could be elegantly intertwined with its role in the cell cycle, where it mediates sister chromatid cohesion. Transcriptional pathways promoting growth are tightly linked to cell division, and it is entirely possible that cohesin and its regulators have central roles in making these links.

### Neuronal development and the transcription of neuronal genes

Neurodevelopmental disorders are among the most conserved features of the cohesinopathies (Deardorff et al., 2007). It is possible that these neurodevelopmental pathologies have a common molecular basis. Several lines of evidence suggest that cohesinopathy proteins influence the Notch signaling pathway, although the exact mechanisms are unknown. A recent study suggested that Esco2 physically interacts with Notch to antagonize Notch signaling, suggesting that one possible mechanism includes direct interaction with Notch receptor(s; Leem et al., 2011).

Our microarray analyses of zebrafish “cohesinopathy” embryos depleted for Rad21, Smc1a, Nipbl, or Esco2 identified conserved regulation of selected gene targets of the Notch signaling pathway. Notably, we found that the *ascl1* gene is downregulated in both *rad21* mutants (Horsfield et al., 2007; Rhodes et al., 2010) and *esco2* morphants (Monnich et al., 2011), as well as Nipbl-depleted embryos and *smc1a* mutants (Table 2; Maren Mönnich, Cristin G. Print, Julia A. Horsfield, unpublished data). In 48 hpf *rad21* heterozygous embryos (which are phenotypically normal), *ascl1* is significantly downregulated (Rhodes et al., 2010), indicating that *ascl1* expression is highly sensitive to even a slight reduction of Rad21 (heterozygotes have 60–70% of wild type *rad21* mRNA levels). Such sensitivity could have high functional significance. *Ascl1* is a potent neuronal lineage-specifying gene, being one of three genes sufficient to convert fibroblasts into iPN cells (Vierbuchen et al., 2010). Furthermore, Pds5b depletion altered *Ascl1* expression and blocked neuronal differentiation in a stem cell model (Denes et al., 2010).

**Table 2:** **Top 20 probe sets affected in zebrafish cohesinopathy microarrays at 24 h post-fertilization (*p* < 0.05)**.

*rad21* Mutant			*smc1a* Mutant			*nipbl* Morphant			*esco2* Morphant		
Affymetrix probe ID	Gene	Log_2_ change	Affymetrix probe ID	Gene	Log_2_ change	Affymetrix probe ID	Gene	Log_2_ change	Affymetrix probe ID	Gene	Log_2_ change
**EXPRESSION LEVELS “UP”**											
Dr.24216.1.S1_at	cki	7.862	Dr.7787.1.S1_at	RPS27	1.056	Dr.21935.1.A1_at	wu:fc84a08	2.927	Dr.17659.1.S1_at		3.483
Dr.5211.1.A1_at		2.374	Dr.4314.1.A1_a_at	wu:fb95d03	0.729	Dr.7787.1.S1_at	RPS27	2.687	Dr.21935.1.A1_at	wu:fc84a08	3.267
Dr.19471.1.A1_at	scamp5	2.116	Dr.15033.1.S1_at		0.647	Dr.13570.1.A1_at	zc3h14	2.591	Dr.11242.1.A1_at	phlda3	3.159
Dr.14046.1.S1_at	UBE2D2	1.918	Dr.2727.1.A1_at		0.646	Dr.11242.1.A1_at	phlda3	2.538	Dr.10334.1.S1_at	casp8	2.926
Dr.4716.2.A1_at	nrarpa	1.813	Dr.4806.1.A1_at	TSTA3	0.642	Dr.11479.1.A1_at	lnx1	2.476	Dr.11479.1.A1_at	lnx1	2.786
Dr.4314.1.A1_x_at	wu:fb95d03	1.635	Dr.14044.1.A1_at	gpr137bb	0.614	Dr.17659.1.S1_at		2.452	Dr.12986.1.A1_a_at	fos	2.636
Dr.25322.1.S1_at	lin7c	1.533	Dr.1190.1.S1_at	anxa1b	0.578	Dr.10334.1.S1_at	casp8	2.406	Dr.23587.1.A1_at	gadd45al	2.632
Dr.17340.1.S1_at	hnmt	1.476	Dr.12502.3.S1_x_at	zgc:171781	0.57	Dr.198.1.S1_at	fst	2.382	Dr.7787.1.S1_at	RPS27	2.628
Dr.7532.1.A1_at	hm:zeh0402	1.397	Dr.5231.1.S1_at	hist2h2l	0.56	Dr.17275.1.A1_at	WHSC2	2.298	Dr.12986.1.A1_at	fos	2.581
Dr.26538.1.A1_at	gpr177	1.388	Dr.5129.1.S1_at	sesn3	0.557	Dr.26372.1.A1_at		2.225	Dr.5925.1.A1_at	wu:fi04f09	2.558
Dr.9457.1.A1_at	egln3	1.287	Dr.1999.1.S1_at	DF	0.55	Dr.542.1.S1_at	mdm2	2.212	Dr.542.1.S1_at	mdm2	2.499
Dr.9478.1.S1_at	cyp1a	1.239	Dr.5820.1.S1_at	ctsll	0.544	Dr.23406.1.S1_at	rpz5	2.168	Dr.11481.1.A1_at	rspo1	2.359
Dr.3432.1.S1_at	capg	1.231	Dr.8453.1.A1_at		0.543	Dr.7768.1.A1_at	shfm1	2.143	Dr.23406.1.S1_at	rpz5	2.219
Dr.24923.2.A1_at		1.204	Dr.15162.3.A1_a_at	pofut1	0.528	Dr.5925.1.A1_at	wu:fi04f09	2.141	Dr.6820.1.A1_at	gtpbp1l	2.173
Dr.18513.2.S1_a_at	sccpdhb	1.164	Dr.5211.1.A1_at		0.519	Dr.10083.1.S1_at		2.126	Dr.2052.1.S1_at	Tp53	2.158
Dr.7738.1.A1_at	aldh18a1	1.157	Dr.3499.3.A1_at	cldni	0.502	Dr.15033.1.S1_at		2.044	Dr.8209.1.S2_at	foxo5	2.098
Dr.5987.1.A1_at	msl1l1	1.139	Dr.15162.1.A1_a_at	pofut1	0.499	Dr.307.1.S1_at	dvr1	2.044	Dr.15033.1.S1_at		2.041
Dr.10735.1.S1_at	caspb	1.107	Dr.2536.1.S1_at	itm2bb	0.499	Dr.14044.1.A1_at	gpr137bb	2.012	Dr.19794.1.A1_at		2.025
Dr.15033.1.S1_at		1.1	Dr.2059.1.A1_at	slc2a2	0.492	Dr.24938.1.S1_a_at	zgc:158463	1.958	Dr.21979.1.A1_at	wu:fc92e10	2.023
Dr.4314.1.A1_a_at	wu:fb95d03	1.088	Dr.18631.2.A1_at	zgc:123295	0.49	Dr.20198.1.S1_a_at	hsp70	1.92	Dr.12986.2.S1_at	fos	1.997
**EXPRESSION LEVELS “DOWN”**											
Dr.5662.1.S1_at	rad21	−3.585	Dr.25729.1.S1_at	CRYGB	−1.511	Dr.5479.1.S1_at	rbp4	−2.219	Dr.20010.14.S1_at		−1.925
Dr.582.1.S1_a_at	cx43	−3.558	Dr.20020.1.S1_at	smc1a	−1.503	Dr.15054.1.S1_at	rbp2a	−1.949	Dr.18151.1.S1_at		−1.512
Dr.10102.2.S1_at	fam212aa	−3.2	Dr.13843.1.S1_at	bhlhe22	−1.485	Dr.5112.1.S3_at	sox11b	−1.813	Dr.16312.1.S1_at	sb:cb25	−1.476
Dr.10102.1.A1_at	fam212aa	−2.432	Dr.4407.1.A1_at	smc1a	−1.322	Dr.22100.1.A1_at	wu:fd13e05	−1.774	Dr.5372.9.S1_s_at	her4.2	−1.401
Dr.1.1.S1_at	myca	−2.271	Dr.15372.1.S1_x_at	CRYGB	−1.22	Dr.8342.1.A1_at	slc6a11	−1.731	Dr.3211.1.A1_at	GRIK2	−1.351
Dr.23067.1.S1_at	myhz1	−2.158	Dr.24771.1.A1_at	smc1a	−0.984	DrAffx.2.105.S1_at		−1.705	Dr.5372.7.A1_x_at	CH73-21G5.3	−1.326
Dr.4812.1.S1_s_at	myhz2	−2.147	Dr.12486.1.S1_at	CLDN11	−0.973	Dr.2778.1.S1_at	STX4A	−1.683	DrAffx.2.105.S1_at		−1.297
Dr.13843.1.S1_at	bhlhe22	−2.118	Dr.10102.2.S1_at	fam212aa	−0.952	Dr.4797.1.S1_at	zgc:123103	−1.682	Dr.5372.1.S1_x_at	her4.2	−1.289
Dr.10343.1.S1_at	atp1a1a.2	−2.074	Dr.22360.1.A1_at	AQP1	−0.879	Dr.21660.1.A1_at		−1.597	Dr.5372.7.A1_at	CH73−21G5.3	−1.246
Dr.22360.1.A1_at	AQP1	−1.857	Dr.19658.1.A1_at		−0.8	Dr.25195.1.S1_at	zgc:158494	−1.563	Dr.20850.1.S1_at	fabp7a	−1.238
Dr.2155.1.S1_at	plk3	−1.797	Dr.4299.1.S1_at	wu:fb83d05	−0.789	Dr.2426.1.S1_at	ambpl	−1.538	Dr.5434.1.S3_at	plp1a	−1.215
Dr.314.1.S1_at	ascl1a	−1.787	Dr.582.1.S1_a_at	cx43	−0.743	Dr.3891.1.A1_at	septin 6	−1.505	Dr.5434.1.S1_at	plp1a	−1.171
Dr.21790.1.A1_at	PCDH18	−1.724	Dr.10102.1.A1_at	fam212aa	−0.724	Dr.5434.1.S1_at	plp1a	−1.502	Dr.2970.1.S1_at	apoea	−1.121
Dr.1280.1.A1_at	cebpd	−1.66	Dr.25173.1.S1_at	smc1a	−0.719	Dr.3004.1.A1_at	AL954182.2	−1.495	Dr.20083.1.A1_at	ccng2	−1.111
Dr.737.1.A1_at	junbl	−1.653	Dr.20850.1.S1_at	fabp7a	−0.666	Dr.18151.1.S1_at		−1.492	Dr.5434.1.S4_at	plp1a	−1.107
Dr.13879.1.A1_at	islr2	−1.638	Dr.6081.1.S1_at	fam212ab	−0.664	Dr.22360.1.A1_at	AQP1	−1.441	AFFX-r2-Bs-dap-3_at		−1.029
Dr.20185.1.S1_at	lpl	−1.637	Dr.21790.1.A1_at	PCDH18	−0.661	Dr.5434.1.S2_at	plp1a	−1.395	Dr.25729.1.S1_at	CRYGB	−1.016
Dr.16053.1.S1_at	hbegfa	−1.577	Dr.3282.1.S1_at	s1pr1	−0.656	Dr.2132.1.A1_at	hao1	−1.388	AFFX-r2-Bs-dap-M_at		−1.012
Dr.318.1.A1_at	ascl1b	−1.558	Dr.314.1.S1_at	ascl1a	−0.655	Dr.13750.1.S1_at		−1.338	Dr.13843.1.S1_at	bhlhe22	−0.997
Dr.20850.1.S1_at	fabp7a	−1.541	Dr.8587.1.A1_at	igfbp1a	−0.652	Dr.4867.1.A1_at	hp	−1.338	Dr.8118.1.A1_at	otpb	−0.995

We also found that certain Notch signaling targets of the *hairy/enhancer of split* family (such as *her4*, *hey1*) were consistently affected in our zebrafish cohesinopathy models (Rhodes et al., 2010), in agreement with cohesin/Nipbl regulation of the (E(spl)-C) in *Drosophila* (Schaaf et al., 2009). In combination with previous gene expression studies from *Drosophila* (Dorsett, 2009), strong evidence supports a link between cohesin-mediated transcription and cell fate in neuronal linages.

In addition to neuronal cell fate, it appears that cohesin together with CTCF could contribute to maintaining neuronal identity. Several studies show that cohesin and CTCF regulate expression of protocadherin genes (Kawauchi et al., 2009; Monahan et al., 2012; Remeseiro et al., 2012b). Cohesin-SA1 binds to the promoter of protocadherin genes and positively regulates their expression (Remeseiro et al., 2012b). Interestingly, CTCF and cohesin were recently found to modulate isoform expression of *Pcdhα* in a mouse neuroblastoma cell line (Monahan et al., 2012), by a mechanism assumed to involve enhancer-promoter communication. Cohesinopathy mutations could therefore have significant consequences for neuronal recognition of “self,” and the capacity to make functional synaptic connections (Dekker, 2012), since protocadherins are key players in these processes (Frank and Kemler, 2002; Esumi et al., 2005).

**Figure 3:**
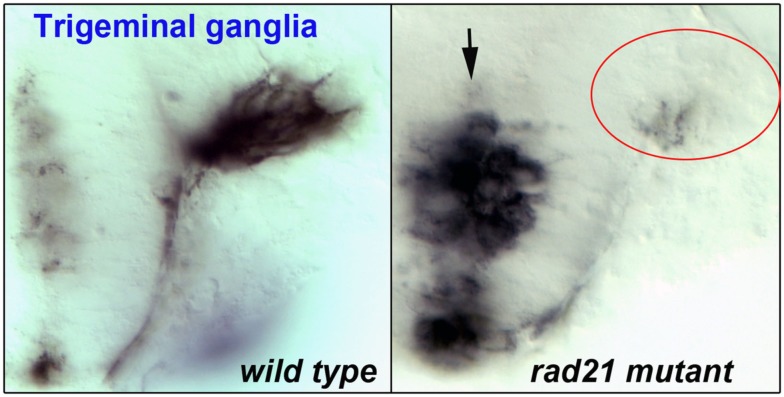
**Flat-mount staining (anti-HNK-1) of trigeminal ganglia in wild type (left) and *rad21* mutant (right) zebrafish embryos**. In *rad21* mutants, central neuronal clumping occurred (arrow), and axons failed to migrate and populate anterior regions (red oval).

Evidence suggests that the widespread disruption of neuronal gene expression found in cohesinopathy mutants results in abnormal behavior and function of neurons. As discussed previously, localized disruption of cohesin subunits causes failure of axon pruning in the *Drosophila* mushroom body (Pauli et al., 2008; Schuldiner et al., 2008). Other model systems have highlighted a role for cohesinopathy proteins in axon pathfinding and/or migration. For example, Mau2, the Scc4 homolog that binds to Nipbl, is necessary for proper axon guidance and migration in *C. elegans* (Seitan et al., 2006). Consistent with a requirement of cohesin for migration, enteric neurons derived from neural crest cells failed to migrate in mice mutant for cohesin subunit *Pds5b* (Zhang et al., 2007). Furthermore, in *esco2* morphant zebrafish, we observed defects that were consistent with abnormal neural crest cell migration (Monnich et al., 2011). In zebrafish mutant for *rad21*, we observed that while the trigeminal ganglia of the brain are specified, the axons clump together rather than extending forward (Figure 3). It is very likely that more subtle defects that are not so easily observed (for example, problems with neuronal connectivity) take place in the central nervous system of cohesinopathy patients and animal models.

Growth, metabolism, and development of the central nervous system appear to be processes that are universally sensitive to cohesinopathy mutations. Much of this pathology is likely to be caused by cohesin’s role in the regulation of gene expression. However, cohesin has another important role in the repair of DNA damage, and its loss is likely to trigger DNA damage checkpoints (Jessberger, 2009; Watrin and Peters, 2009). Activation of cell cycle checkpoints by cohesin depletion may represent additional biological processes contributing to cohesinopathies.

## Cohesinopathy Genes, DNA Damage, and Cell Cycle Checkpoints

When damaged DNA is detected, cells respond by coordinating cell cycle arrest, DNA repair, and programmed cell death (Ciccia and Elledge, 2010). The crucial roles of cohesin and its regulators in DNA damage repair have been recently and comprehensively reviewed elsewhere (Dorsett and Strom, 2012; Wu and Yu, 2012). Inability to repair DNA and proceed through the cell cycle is accompanied by activation of cell cycle checkpoints, followed by cell death in the absence of repair.

Interestingly, mutations in other genes responsible for the DNA damage response underlie human syndromes with phenotypes that overlap the cohesinopathies (Ciccia and Elledge, 2010). Overlapping phenotypes include microcephaly, growth defects, neurological disorders, and facial/skeletal dysmorphology. These features are among the most conserved between the cohesinopathies, and raise the possibility that defects in the DNA damage response pathway might contribute to the etiology of cohesinopathy syndromes. In support of this, a patient with a mutation in a gene encoding the DNA helicase DDX11/ChlR1 had microcephaly, premature sister chromatid separation, and genome instability. This patient had features of both Fanconi Anemia (associated with other DNA helicases involved in DNA damage repair, XPD, and FANCJ) and RBS, in which *ESCO2* is mutated. The syndrome, known as Warsaw Breakage Syndrome, is considered to reside at an interface between DNA damage repair and sister chromatid cohesion (van der Lelij et al., 2010).

It is possible that the CoAT ESCO2 has a particularly crucial role in DNA damage repair, since mutations in ESCO2 appear to resemble mutations in DNA damage repair pathways more than the other cohesinopathies do. Indeed, ESCO2-depleted cells are hypersensitive to DNA damaging agents such as Mitomycin C (van der Lelij et al., 2009; Whelan et al., 2012a). Acetylation of SMC3 is necessary for S phase checkpoint activation and cell survival (Kim et al., 2010a), which might explain the absolute requirement for ESCO2 at this stage of the cell cycle.

Other cohesinopathy mutations also have potential to compromise DNA damage repair. Mice heterozygous for a *Rad21* null mutation are hypersensitive to IR, and exhibit problems with integrity and maintenance of the gastrointestinal tract and hematopoietic system post-irradiation (Xu et al., 2010). In humans, patients with *RAD21* mutations also have impaired DNA damage repair (Deardorff et al., 2012b), and knock down of *RAD21* sensitizes breast cancer cells to chemical agents that damage DNA (Atienza et al., 2005; Xu et al., 2011). Therefore, full dosage and function of the *Rad21* gene is crucial for DNA damage repair. In addition, depletion of SMC1 sensitizes HeLa cells to DNA damage (Bauerschmidt et al., 2010). Interestingly, the cohesin regulator PDS5B (APRIN) and the cohesin subunits RAD21 and SMC3 were recently found to associate with the BRCA2 protein. PDS5B appears to have an essential function in both the DNA damage response and homologous recombination (Brough et al., 2012).

It is not clear to what extent DNA damage repair defects contribute the pathology of cohesinopathies (Dorsett and Strom, 2012), but evidence suggests that most cohesinopathy mutations are likely to impact on the cell cycle in intra-S and G2 phases, when DNA damage repair takes place. Insufficiency of DNA damage repair should lead to checkpoint activation and cell death, potentially resulting in a paucity of cells for adequate development. However, many cohesinopathy mutations give rise to altered transcription of developmental regulators rather than cell cycle phenotypes, raising the question of how distinct outcomes arise from mutations in proteins with a related function in the cell cycle.

## A Model to Explain Diverse Cohesinopathy Phenotypes

**Figure 4:**
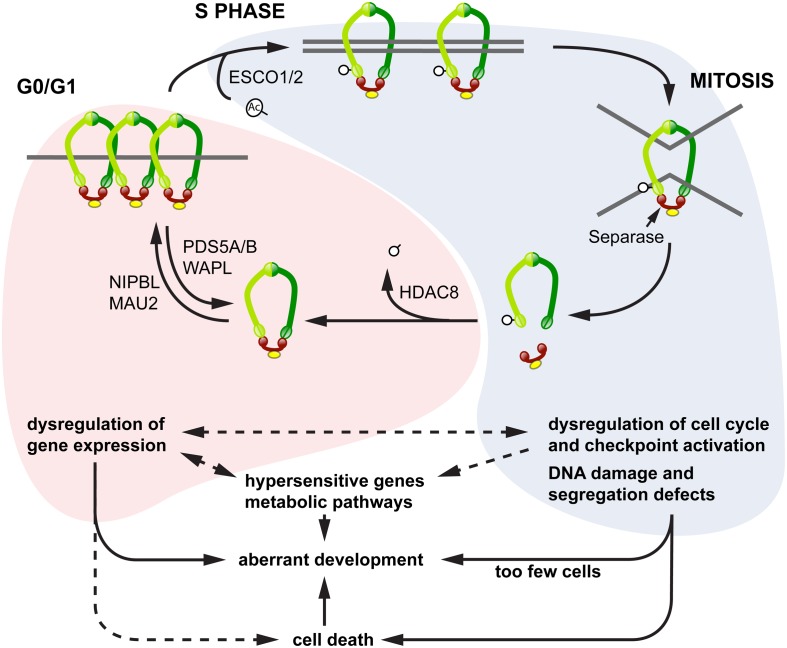
**Model for diverse cohesinopathy phenotypes**. In interphase (red shading), cohesin binding to chromatin is dynamic, with varying residency times. Interphase cohesin binding is likely to be cell type-specific and to contribute toward regulating developmental genes. Mutations in cohesin subunits and their key interphase regulators (e.g., Nipbl, Hdac8) primarily impact on the regulation of gene expression, including transcriptional regulation of growth pathways. This results in syndromic developmental defects that derive from dysregulated transcription, with the possibility of cell death as a contributing factor. From S phase to G2/M (blue shading), the overriding function of cohesin involves sister chromatid cohesion and DNA damage repair. Key regulators in this process include the CoAT ESCO2 and other DNA damage repair proteins. Mutations in these regulators result in chromosome segregation defects, genomic instability, and cell death. Increased cell death and reduced cell proliferation results in too few cells to make up body structures, leading to a different class of developmental defects and dysregulation of metabolic pathways. Transcription of a small subset of hypersensitive genes, including some in the Notch signaling pathway, appears to be sensitive to both interphase and S/G2 modes of cohesin binding.

We propose a model to explain the diverse phenotypes observed downstream of cohesinopathy genes, in which different phenotypes emerge according to the “phase” of the cohesin cycle that is *most* affected by a particular cohesinopathy mutation in a given population of cells (Figure 4). In this model, mutations affecting cohesin loading and its residency times on chromatin in interphase have a higher potential to influence the regulation of gene expression, since this function can be exquisitely sensitive to cohesin dose. Alternatively, mutations affecting the “cohesive” form of cohesin have more potential to impact on cell division, DNA damage repair, and cell cycle checkpoints. The consequences are that the latter mutations will affect sister chromatid cohesion, and initiate cell death pathways. Shared phenotypes such as microcephaly, craniofacial defects, and cognitive impairment are likely to lie at the interface between these two pathways.

It is important to note that cohesin subunits and the cohesin loading kollerin complex participate in *all* cohesin-related processes, including transcription regulation during interphase, chromatid cohesion during S phase, and DNA damage repair. Therefore mutations in genes encoding these proteins have potential to disrupt all the processes shown in the model (Figure 4). However, diverse outcomes from different cohesinopathy mutations could result if certain processes have differential sensitivity to loss of cohesin components and regulators, in distinct cell populations.

For example, zebrafish embryos zygotic null for *rad21* contain heavy maternal loading of Rad21 protein and are able to develop for about 20 h before cell cycle deficiencies halt growth. However, well before cell cycle defects have any impact, *rad21* null embryos fail to activate *runx1* expression in the hematopoietic mesoderm (Horsfield et al., 2007). Thus, there is a threshold level of cohesin essential for *runx1* expression that is below the level necessary to sustain cell division. The primary impact of suboptimal levels of Rad21 is that of altered gene expression in a subpopulation of cells, and the secondary impact of cell cycle arrest is not observed until Rad21 levels are further depleted. Radiation sensitivity observed in *Rad21* heterozygous mice (Xu et al., 2010) and in cells of patients with compromised RAD21 function (Deardorff et al., 2012b) indicates other functions of Rad21 are also dose-sensitive.

In summary, the phenotypic outcome of cohesinopathy mutations may differ between cell populations and in any given cell population, depend upon the degree of sensitivity of gene expression to cohesin levels, the requirement for cell proliferation, and the presence of environmental stressors such as DNA damaging agents. There is likely to be significant overlap in these contributing factors to cohesin-related developmental disorders.

## Conclusion

Although considerable progress has been made over the last 10 years in the understanding of cohesin function in the cell cycle, transcription, and human developmental disease, important questions remain. How is the transcriptional role of cohesin coordinated with its role in genome organization, cell division, and DNA repair? Why do some cohesinopathy mutations lead to developmental gene dysregulation, while others lead to chromosome segregation defects and cell death? Human syndromes and animal models have potential to lend important insight into the integration of cohesin functions in cell division and development. Continued research will be vital for understanding the pathology of cohesinopathy syndromes, and the development of future potential for clinical management or therapy.

## Conflict of Interest Statement

The authors declare that the research was conducted in the absence of any commercial or financial relationships that could be construed as a potential conflict of interest.
